# Dynamical Principles of Emotion-Cognition Interaction: Mathematical Images of Mental Disorders

**DOI:** 10.1371/journal.pone.0012547

**Published:** 2010-09-21

**Authors:** Mikhail I. Rabinovich, Mehmet K. Muezzinoglu, Irina Strigo, Alexander Bystritsky

**Affiliations:** 1 BioCircuits Institute, University of California San Diego, La Jolla, California, United States of America; 2 Psychiatry Department, University of California San Diego, La Jolla, California, United States of America; 3 Department of Psychiatry and Biobehavioral Science, University of California Los Angeles, Los Angeles, California, United States of America; Mount Sinai School of Medicine, United States of America

## Abstract

The key contribution of this work is to introduce a mathematical framework to understand self-organized dynamics in the brain that can explain certain aspects of itinerant behavior. Specifically, we introduce a model based upon the coupling of generalized Lotka-Volterra systems. This coupling is based upon competition for common resources. The system can be regarded as a normal or canonical form for any distributed system that shows self-organized dynamics that entail winnerless competition. Crucially, we will show that some of the fundamental instabilities that arise in these coupled systems are remarkably similar to endogenous activity seen in the brain (using EEG and fMRI). Furthermore, by changing a small subset of the system's parameters we can produce bifurcations and metastable sequential dynamics changing, which bear a remarkable similarity to pathological brain states seen in psychiatry. In what follows, we will consider the coupling of two macroscopic modes of brain activity, which, in a purely descriptive fashion, we will label as cognitive and emotional modes. Our aim is to examine the dynamical structures that emerge when coupling these two modes and relate them tentatively to brain activity in normal and non-normal states.

## Introduction

The view that the brain is an active system that entails the acquisition and maintenance of information for responding to environmental events has a long history [1]–[4]. On a coarse grain level of description, mental brain activity can be represented by a dynamical model as the activity of a complex nonequilibrium system [5], [6]. In spite of the fact that the brain is a noisy place, i.e., individual responses of single neurons to stimuli are highly variable, the cooperative activity of a large number of neurons is robust against noise and reproducible [6], [7]. The principles of mental activity and, in particular those regarding the cognition-emotion interaction that we are going to discuss in this paper, are based on experimental observations supporting the following statement: the human brain is intrinsically organized into active, interactive functional networks and its effective coarse grain activity can be described by a dynamical model.

Traditional efforts in modeling dynamical phenomena in the brain are predominantly based on the premise that dynamical systems tend to converge to stable fixed points or dynamical states (limit cycles or strange attractors) where the density of all flows (matter, energy, or information) are balanced and do not change (see, for example, [8]). Active neuronal networks in some specific conditions - (with symmetric reciprocal interactions) give rise to a convergent mental activity involving multiple attractors [9], [10]. There may be some cognitive activities, such as associative memory [11], which fits the attractor-oriented description. However, mental computing with attractors generally limits the use of complex dynamical networks. Once the attractor (or its vicinity) is reached, the “dynamical” nature of the brain becomes irrelevant; therefore, when attractors mark the terminal states of mental process, this behavior could be formulated equally effectively by an algebraic cause-response mapping. Furthermore, this scheme overlooks the qualities of the (transient) path from the initial condition to the attractor, an important phase where the brain could exploit its remarkable repertoire of behaviors. Thus, confining dynamical models of the brain with global and symmetric coupling is not only unrealistic, but also rules out a continuum of opportunities for modeling and understanding mental activity. In this paper, we propose an alternative paradigm, i.e., stable transient dynamics, which can be implemented in the realistic case of non-symmetric connections between interacting mental agents.

**Figure 1:**
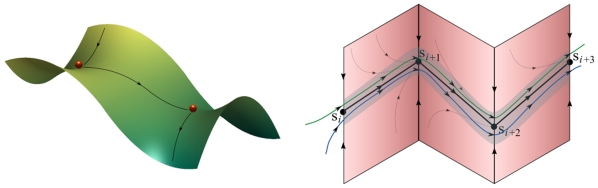
Heteroclinic chain. (Left panel) The simplest sequence of two metastable states (i.e., saddles), where the unstable separatrix of the first saddle is owned by the stable separatrix of the second. (Right panel) The sequence can be (structurally) stable, i.e., attractive for any point in its vicinity marked by the dark stripe.

One of the most intriguing components of mental transients is the metastable state, which provides the structural stability of transient behavior [12]. Metastability is a system-level phenomenon, which is becoming increasingly popular in neuroscience for the elucidation of human information processing and pattern recognition. Metastability imposes semi-transient signals in the brain, which persist briefly and differ from the usual equilibrium state [13]. The metastable activity of the cortex can also be inferred from behavior [14]. Metastability is a principle that describes the brain's ability to make sense out of seemingly random environmental cues [15], [16]. The existence of mental metastable states, supported by interactions observed among diverse brain centers or neuron groups [17]–[21], is the result of self-organization in very complex neuronal systems. The temporal order of metastable states is determined by the functional connectivity of the underlying networks and their causality structure [22]. The mathematical image of a metastable state is a saddle set in the working (state) space of the brain; the transition between these saddles occurs via unstable separatrices connecting them (see Fig. 1). There is a substantial experimental support [13], [23], [24] (also outlined below) that metastability and transient dynamics are key phenomena in brain dynamics.

Neuroimaging and multi-electrode recording experiments reported in the last decades have shown that various brain functions and psychiatric disorders are represented by different spatio-temporal brain activity patterns. Such patterns are a sequence of metastable states. Each metastable state is the result of a coordinated interaction between emotional, cognitive and perceptional *modes*; that is, a set of neuronal groups in different parts of the brain, which are self-organized for the execution of a specific mental function. The modes interact with each other according to the following general principles: (1) they compete for limited mental resources (attention, memory), (2) they demonstrate a stable and reproducible dynamics that is sensitive to the informational signals; and (3) their dynamics is transient and evolves via metastable states. Based on these principles, we introduce a dynamical model of interaction of modes that we label as emotional and cognitive. The model has a form of coupled generalized Lotka-Volterra kinetic equations and has demonstrated a spectrum of qualitatively different activity patterns and bifurcations depending on the value of a moderate number of control parameters. We wish to examine the dynamical structures that emerge when coupling cognitive and emotional modes, and relate them tentatively to brain activity in normal and pathological states (see, for example, [25], [26]).

The paper is organized as follows: first, we introduce a canonical model for emotion- cognition mode interaction based on the above-mentioned main principles. Then we analyze its dynamical features and the corresponding dynamical objects in phase space which correlate with specific types of mental activities in healthy and disordered brains. We will illustrate the model abilities on examples: the spontaneous “resting-state” dynamics characterized by low-frequency pulsations, and the dynamics of anxiety disorders, such as panic attack and obsessive-compulsive disorder (OCD). We also analyze a “cognitive performance – arousal” interaction focusing on a hysteresis phenomenon.

## Materials and Methods

### Mental Modes: Dynamical Variables and Phase Space

Numerous attempts have been made to quantify cognition, i.e., problem solving by information processing, and emotion, i.e., spontaneous motivation and subsequent implementation of a behavior. Being directly related to the processing of auxiliary information, cognition has attracted relatively more attention compared to emotion in these efforts, particularly in the form of decision-making tasks [27], [28]. Although several tests aiming to assess emotions exist (see, for example, [29]), these have often been confounded by concomitant cognitive processes, such as appraisal [30], [31], decision making [32], or memory [33], [34].

Which variables we needed to describe the evolution of the emotional and cognitive modes while capturing their functional complexity? To answer this question, we look at an example of complex systems in non-living nature, such as turbulent flows [35]. A macroscopic description of turbulence can be made using equations for coarse-grain liquid particles; the micro details of the molecular dynamics are irrelevant. Of course, these micro details are important because they determine the parameters of the macroscopic model. However, the basic coarse-grain equations are much simpler and transparent. Although the situation regarding mental dynamics is much more complex, we can still apply the turbulent flow analogy. Using this approach, a neural mass model has been suggested for the simulation of cortical activity [36]–[38]. Our approach – based on mental mode interaction - is also coarse-grain.

The dynamical variables, i.e., the amplitude of the different mental modes describing emotion, cognition, and mental resources consumed by them, form a joint state space (or phase space). We assume that a specific cognitive activity (e.g., appraisal or sequential navigation) can be described by the interaction of a finite number (N) of cognitive modes and that such interaction is both reproducible and distinguishable over time. Thus, a spatio-temporal movie of such cognitive activity can be captured, for example, by a series of functional Magnetic Resonance Imaging (fMRI) snapshots taken at consecutive times while the subject engages in a specific cognitive task. There are several efficient ways to extract the modes from the experimental data, e.g., by principal or independent components analyses of temporal brain activity [39]–[41]. Thus, the cognitive activity at time *t* can be represented as 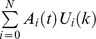, where *U_i_*(*k*) is a function that characterizes the averaged relative activities of *k* participants of a distributed neuronal set that forms the *i*-th cognitive mode, and *A_i_*(*t*) is the level of activity of this mode at time *t*. Some of the cognitive modes, can be responsible for the interaction with emotion, for example, arousal and generation of any given coping strategy (see also [42]). The number *N* of these modes depends on the level of details that we wish to describe. Emotional activity can be represented in the same way - 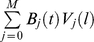, where *B_j_*(*t*) are dynamical variables and *V_j_*(*l*) is a function that characterizes the structure of the *j-th* emotional mode. The ensemble of emotional modes includes both positive and negative emotions in our model. Resources are represented in a similar manner.

### A Canonical Model of the Mental Dynamics

In the last few years, the nonlinear dynamical theory has formulated the concept of stable transients that are robust against noise, yet sensitive to the external signal [12], [24]. The mathematical object that corresponds to such stable transients is a sequence of the metastable states that are connected by special trajectories named separatrices (see Fig. 1). Under proper conditions (as outlined in the Appendix), all trajectories in the neighborhood of the metastable states that form the chain remain in their vicinity, ensuring robustness and reproducibility in a wide range of the control parameters. Because such sequence is possibly the only dynamical object that satisfies the dynamical principles that were formulated above, we assume that from the dynamical point of view, mental activity is also a sequence of the metastable states.

The following is the formulation of the desired features of the model: the model must be dissipative with an unstable trivial state (origin) in the phase space and the corresponding linear increments must be stabilized by the nonlinear terms organized by self- and mutual-inhibition (mode competition); the phase space of the system must include the metastable states that represent the activity of an individual mode when other modes are passive; and finally these metastable states must be connected by separatrices to build a sequence. Well-known rate models in neuroscience satisfy these conditions in some regions of the control parameter space [43], [44]. Thus, the canonical model describing the mode dynamics employs the nonlinear rate equations:
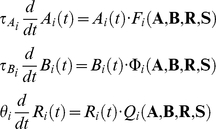
(1)where *A_i_≥0*, *i* = 1,…,*N*, represents the cognitive modes, *B_i_*, *i* = 1,…,*M*, represents the emotional modes and *R_i_*, *i* = 1,…,*K*, represents the resources consumed by these mental processes. *F_i_*, Φ_i_, and *Q_i_* are functions of *A_i_*, *B_i_*, and *R_i_*, respectively. The collections of *N* cognitive modes, *M* emotional modes, and *K* resource items are encapsulated in **A**, **B**, and **R**, respectively. When initiated properly, this set of equations ensures that all the variables remain non-negative. The vector **S** represents the external or/and internal inputs to the system and τ*_A_*, τ*_B_*, and *θ* are the time constants.

First, let us apply the model (1) to just one form of the mental activity when cognition-emotion interaction is negligible. Let us imagine a situation where cognition changes over time while emotion remains more or less constant over time. Keeping in mind that the competition between the different modes of cognitive activity can be described in the simplest form of functions on the right side of equation (1), i.e., *F*(*A*,*S*) being linear, we can present the first set of equations (1) in a standard form of a generalized Lotka-Volterra (GLV) model [45]:
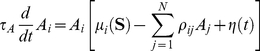
(2)Here *μ_i_*(**S**) is the increment that represents both intrinsic and external excitation, *ρ_ij_* is the competition matrix between the cognitive modes, *η*(*t*) is a multiplicative noise perturbing the system, **S** is the input that captures the sources of internal or external effects on the increment. A similar model can describe the competition between the emotional modes when cognition does not influence the emotion.

The model (2) has many remarkable features, which we will use to build and understand the canonical model; depending on the control parameters, it can describe a vast array of mental behaviors. In particular, when connections are nearly symmetric, i.e., *ρ_ij_≈ρ_ji_*, two or more stable states can co-exist, yielding multi-stable dynamics where the initial condition determines the final state. When the connections are strongly non-symmetric, a stable sequence of the metastable states can emerge [12] (see Fig. 1). The non-symmetric inhibitory interaction between the modes helps to solve an apparent paradox related to the notion that sensitivity and reliability in a network can coexist: the joint action of the external input and a stimulus-dependent connectivity matrix defines the stimulus-specific sequence. Dynamical chaos can also be observed in this case [46]. Furthermore, a specific kind of the dynamical chaos, where the order of the switching is deterministic, but the lifetime of the metastable states is random, is possible [47]. Similar “timing chaos with serial order” has been observed *in vivo* in the gustatory cortex [23].

For the model (2), the area in the control parameter space with a structural stability of the transients has been formulated in [12] (see the Appendix in File S1).

Describing the interaction between the cognitive modes, emotional modes, and the resources consumed by these mental processes, we are particularly interested in a structurally stable transient mental activity, which can effectively describe the reproducible activation patterns during normal mental states and identify specific instabilities that correspond to mental disorders. Based on the GLV model (2), we introduce the system (1) as follows:
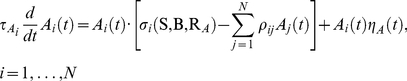
(3)

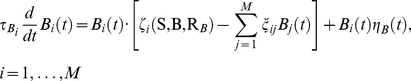
(4)

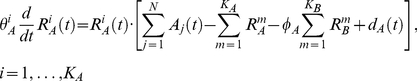
(5)

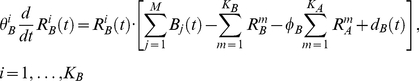
(6)


The proposed model (3)–(6) reflects a mutual inhibition and excitation within and among these three sets of modes (see Table 1). These modes depend on the inputs through parameter **S** (that may represent, for example, stress, cognitive load, physical state of the body). The variables *R^i^_A_* and *R^i^_B_* characterize the *K_A_* and *K_B_* resource items that are allocated to cognition and emotion, respectively. The vectors **R** *_A_* and **R** *_B_* are the collections of these items that gate the increments of the cognitive and emotional modes in competition. The characteristic times *θ* of the different resources may vary. The coefficients *φ_A_* and *φ_B_* determine the level of competition between cognition and emotion for these resources. Each process is open to the multiplicative noise denoted by *η* and *d* terms in the equations.

**Table 1:** Model parameters and their values used the simulations.

*Parameter*	*Role*	*Range of values in simulations*
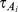	Time constants for cognitive modes	1e-2 to1e-1
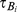	Time constants for cognitive modes	1e-2 to 1e-1
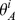 , 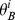	Time constants for resource dynamics	1
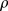 , 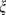	Competition matrices – inducing metastable state sequence	Selected according to the inequalities in Appendix, assuming all increment values equal unity
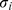	Increments to the cognition modes – locating the metastable states	Dependent variable within 0 and 1: proportional to exogenous input *S*, inversely proportional to either a specific emotion mode, or the total emotional activity, i.e., Σ *B_i_*
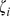	Increments to the emotion modes	Dependent variable within 0 and 1: either a constant of 1 or inversely proportional to Σ *A_i_*
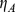 , 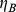 , *d_A_*, *d_B_*	Noise components	Uniform random terms within 0 and *u*, where u is set between 1e-6 and 1e-3
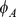 , 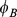	Regulate the resource modes competition	Constants set to 1, except in hysteresis simulation, where 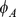 = 0.33 and 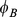 = 1.0

The values of the increments σ*_i_* and ζ*_i_* depend on the stimuli and/or the intensity of the emotional and cognitive modes, respectively. The only design constraint that we can impose on the increments σ*_i_* and ζ*_i_* is that they must stay positive.

Three types of interactions are described by the model (3)–(6): (i) a competitive interaction within each set of modes; (ii) the interaction through excitation (increments); and (iii) the competition for resources. For the latter, which occurs via variables *R_A_* and *R_B_*, one only needs a proper selection of the parameters *φ_A_* and *φ_B_*. Despite the computational simplicity in their selection, they appear to be highly individual- and task-specific. The time constants are the decisive parameters of the model and should be determined *ad hoc* experimentally. The values of the control parameters of the model, which ensure stability of the transients (for normal behavior), can be obtained from the inequalities that describe the ratio between compressing and stretching of the phase volume in the vicinity of the metastable states [12]. The effective number of the parameters can be much smaller than that listed in the model.

The brain imaging data available today does not reveal the detailed structure of the modes and values of parameters, most importantly, the connectivity matrix to specify the model for different mental functions and disorders; therefore, a complete theoretical description and prediction is not possible today. In spite of this, the model has a large dynamical repertoire and has just enough number of parameters to demonstrate possible behaviors and transitions among them, i.e., bifurcations. This capability, together with demonstrated success in representing some key phenomena observed in the real brain, is a valuable qualitative prediction by itself and can be useful for understanding the origin of observed mental phenomena such as depression, working memory, and decision making in a changing environment [48], [49].

The dynamical objects in the phase space of the model representing mental processes are influenced by the intrinsic brain dynamics and by the external stimuli. For example, during sequential decision-making, sequential working memory or navigation, the image of the cognitive dynamics is a stable transient, while other common cognitive activities, such as those pertaining to music [50] or linguistic functions [51] can be represented by the recurrent dynamics. Emotion can also demonstrate a whole range of the dynamical behaviors: transient regimes similar to cognitive ones, recurrent regular or irregular recurrence dynamics corresponding to mood changes; and long lasting equilibria associated with clinical cases of deep depression or constant excitement.

## Results

### Modulation Instability: possible dynamical origin of low-frequency resting-state oscillations

**Figure 2:**
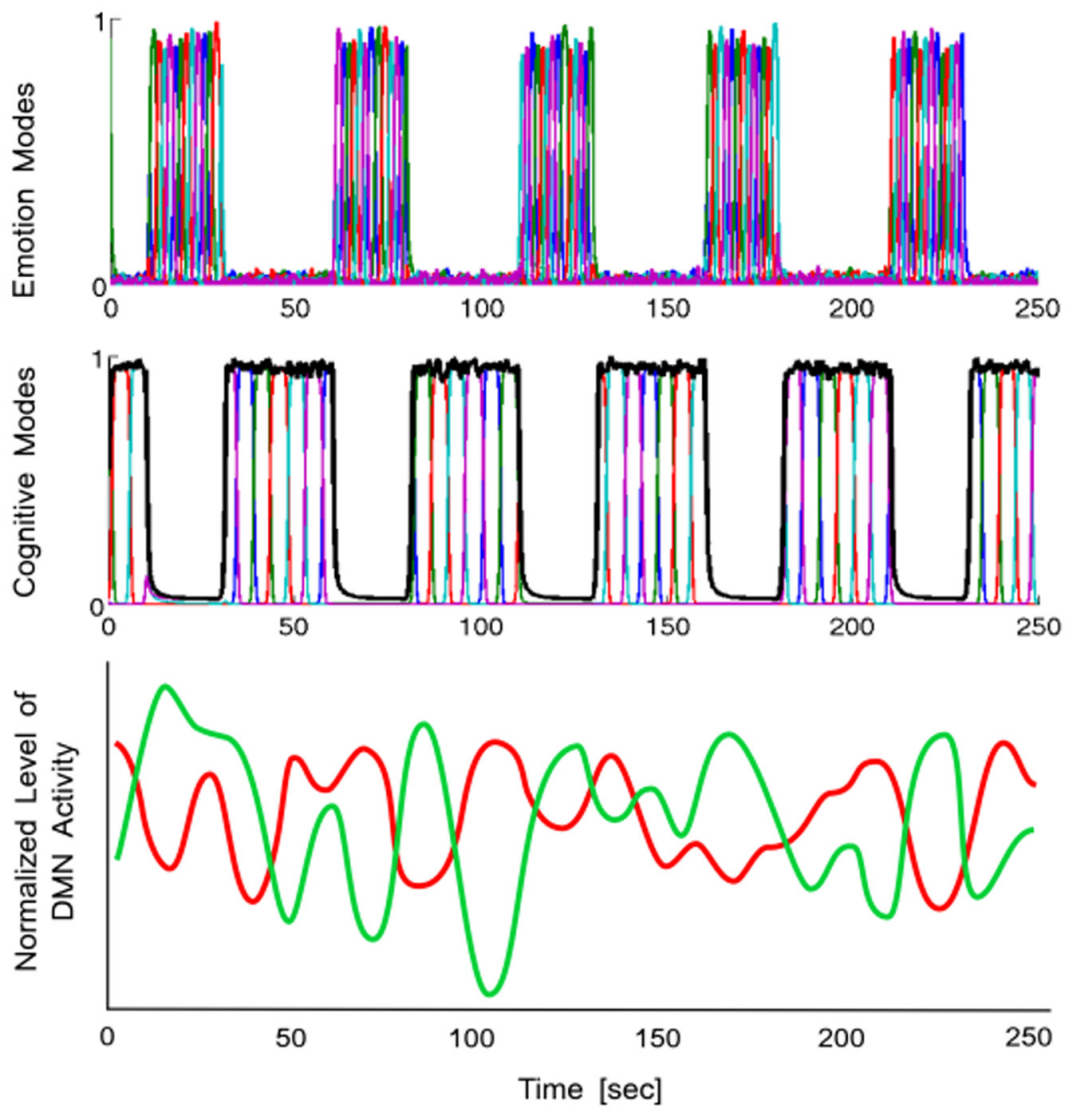
Anti-phase Low-Frequency Oscillation of mental activity. In the resting state, it is a result of two groups of default modes competition – modulational instability (S is constant, N = M = 5 in the model (3)–(6)). The black envelope on the middle plot is the total cognitive activity as predicted by the model. Its competition with the emotion modes (top row) results in a pulsation as observed in many EEG and fMRI studies. The bottom figure, reconstructed based on the data presented in [53], shows one such observation in the brain's resting state.

Let us first analyze spontaneous mental dynamics in a stationary environment where the brain is not engaged in a particular cognitive function, i.e., resting-state brain dynamics. Such resting state is related to the dynamics of the Default-Mode Network (DMN), which is a set of specific brain regions whose activity is predominant during the resting state [52]. We have chosen the control parameters of the model (3)–(6) in the area of the control parameter space where the basic dynamics of both cognitive and emotional modes demonstrate simple rhythmic activity (oscillations with a characteristic time scale of 2–3 sec). Such “independent” emotional and cognitive activity has been observed during weak competition. When competition becomes larger than the critical value, this simple rhythmic activity becomes unstable due to modulation instability, thereby a stable limit cycle appears on the phase plane of the mean activity: 
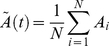
 and 
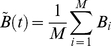
. This modulation instability leads to stable Low Frequency Oscillations (LFO), as shown in Fig. 2 (see also [53]). The averaged-in-time time series indicates that the observed robust modulation process is close to the quasi-periodic LFO.

An increasing number of EEG and resting state fMRI studies in both humans and animals indicate that spontaneous low frequency fluctuations in cerebral activity at 0.01–0.1 Hz represent a fundamental component of brain functioning. In particular, resting state fMRI measures show stable properties of LFO (see Fig. 2), the nature of which are only beginning to be uncovered and have produced a lot of debates. Observed LFO in fMRI, for instance, could simply be due to the band-pass filtering effect of the hemodynamic response function. In general, the LFO fluctuations observed with fMRI are not the same as the underlying neuronal fluctuations because they have been passed through a hemodynamic response function. However, some data [54]–[57] supports the hypothesis that LFO are correlated with the network's activity, i.e. modes cooperative dynamics (e.g. due to their modulation or synchronization). The modulation instability that we have observed in the computer experiments discloses a plausible dynamical origin of low frequency mental mode dynamics in the resting states, possibly related to the “cortical-subcortical cross-talk” [58]. Importantly, discovering changes in the resting state dynamics in various psychiatric disorders may provide a new tool for the diagnosis of psychopathology or for identifying individual variations in physiological arousal [59]–[62].

### Psychopathology and Emotional Instability

Let us consider anxiety disorders that have been associated with abnormal activity in distinct brain networks or modes including hippocampus anterior cingualte, insula, basal ganglia, and some others [63], [64]. Although there is large symptomatic overlap between different anxiety disorders, each disorder can be characterized by a specific quality of anxiety dynamics, i.e., specific emotional instability, and, thus, can be represented by its own dynamical object in the phase space of the canonical model with appropriate value of the control parameters.

Below we will explain a possible dynamical description for panic attack and Obsessive-Compulsive Disorder (OCD). From the dynamical theory point of view, normal emotion- and cognition-related activities occur between pathological but stable states (like deep depression, coma, etc.) and “the edge of chaos” which is also a pathological. Psychopathology, like panic attack and OCD, is associated with irregular dynamical behavior and is often more consistent with chaotic dynamics (see, for example [65]–[68]). Mathematical images of such dynamics are transient chaos or strange attractors [69].

Panic attacks occur in many different types of anxiety disorders. They have a sudden onset and typically peak within 10 minutes. There are several interesting views and models of panic attack in the literature [70], [71]. In particular, Callahan and Sashi [71] have suggested an emotion model based on the catastrophe theory which represents some qualitative characteristics of pathologic affective response. While such perspectives are useful for the integration of biophysical formulations of the interaction between the biology and social experience [72], our view is that any description of emotion as a stand-alone phenomenon, isolated from other players of mentality, would be limited in utility.

**Figure 3:**
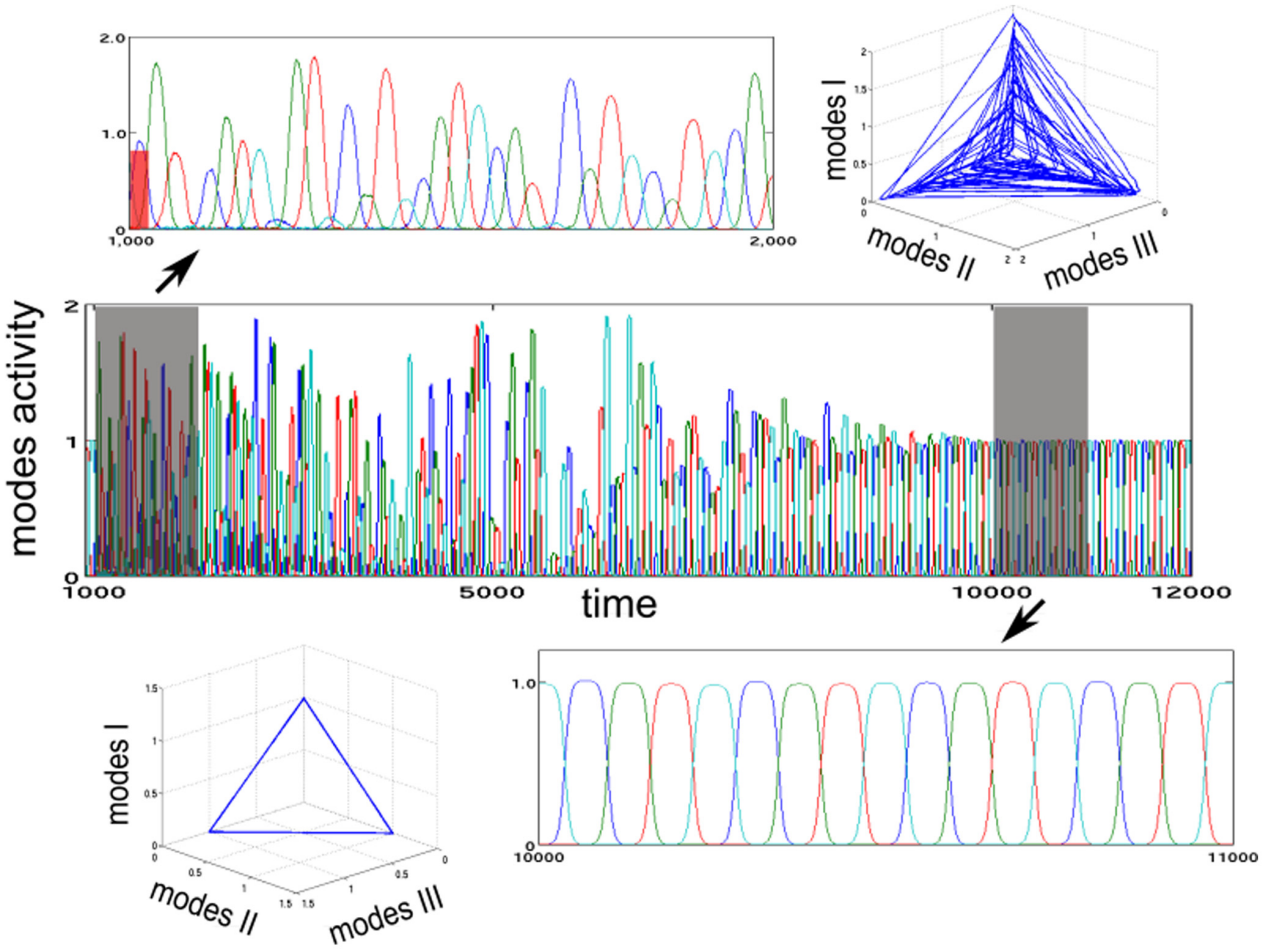
A simulation of a panic disorder on the emotional or the cognitive mode evolution. The panic attack arises due to an external disturbance (represented as an instantaneous kick marked by a red ticker in the inset above) at time *t* = 1000*s*. The attack is characterized by an irregularity in both the cognition and the emotion dynamics. The magnitudes (thus, the switching order) of each group of modes are non-deterministic during this period. In this example, the system returns to its regular pace after some period (i.e., *t*>10,000 *s*). The top row shows an early phase of the irregular activity due to the attack both in time and in a phase portrait. The situation after turning to “normal” is shown on the bottom row.

Based on equations (3)–(6), we modeled the interaction of modes that can be related to panic attack with modes of sequential cognitive activity (- e.g., sequential decision-making). When the interaction cognition with emotion is negligibly small, the dynamical object representing such cognitive activity in the phase space is a robust periodic or quasi-periodic sequence of the metastable states. The model reproduces this activity when the (σ,ρ) pair satisfies certain stability conditions (see Appendix) to construct a stable chain of metastable states in the cognitive subspace of the joint phase space. Suppose now that the interaction function σ(**B**) is non-monotonic and large for some **B** components, and the resource competition is mild (i.e., *φ*_A_ and *φ*_B_ are small). As can be seen in Fig. 3, the dynamical object corresponding to the temporal interaction between emotion and cognition during a panic attack is transient chaos, i.e., both the cognitive activity and the emotion become unpredictable for a finite time (see the time series in Fig. 3). Such chaotic mental activity can be quantitatively characterized by the maximal transient Kolmogorov-Sinai entropy, which is approximately 0.34 (during the panic attack period) in our example.

The OCD is a type of an anxiety disorder that traps people in the endless cycles of repetitive feelings, unwanted thoughts and unwanted repetitive acts which the sufferer realizes are undesirable but is unable to resist – compulsive rituals [73], [74]. The compulsive rituals characteristic of OCD are performed in an attempt to prevent the obsessive thoughts or make them go away. Although the ritualistic behavior may make the anxiety go away temporarily, the person must perform the ritualistic behavior again when the obsessive thoughts return. People with OCD may be aware that their obsessions and compulsions are senseless or unrealistic, but they cannot stop themselves. An attractor-based description of the OCD has been attempted recently in [75].

**Figure 4:**
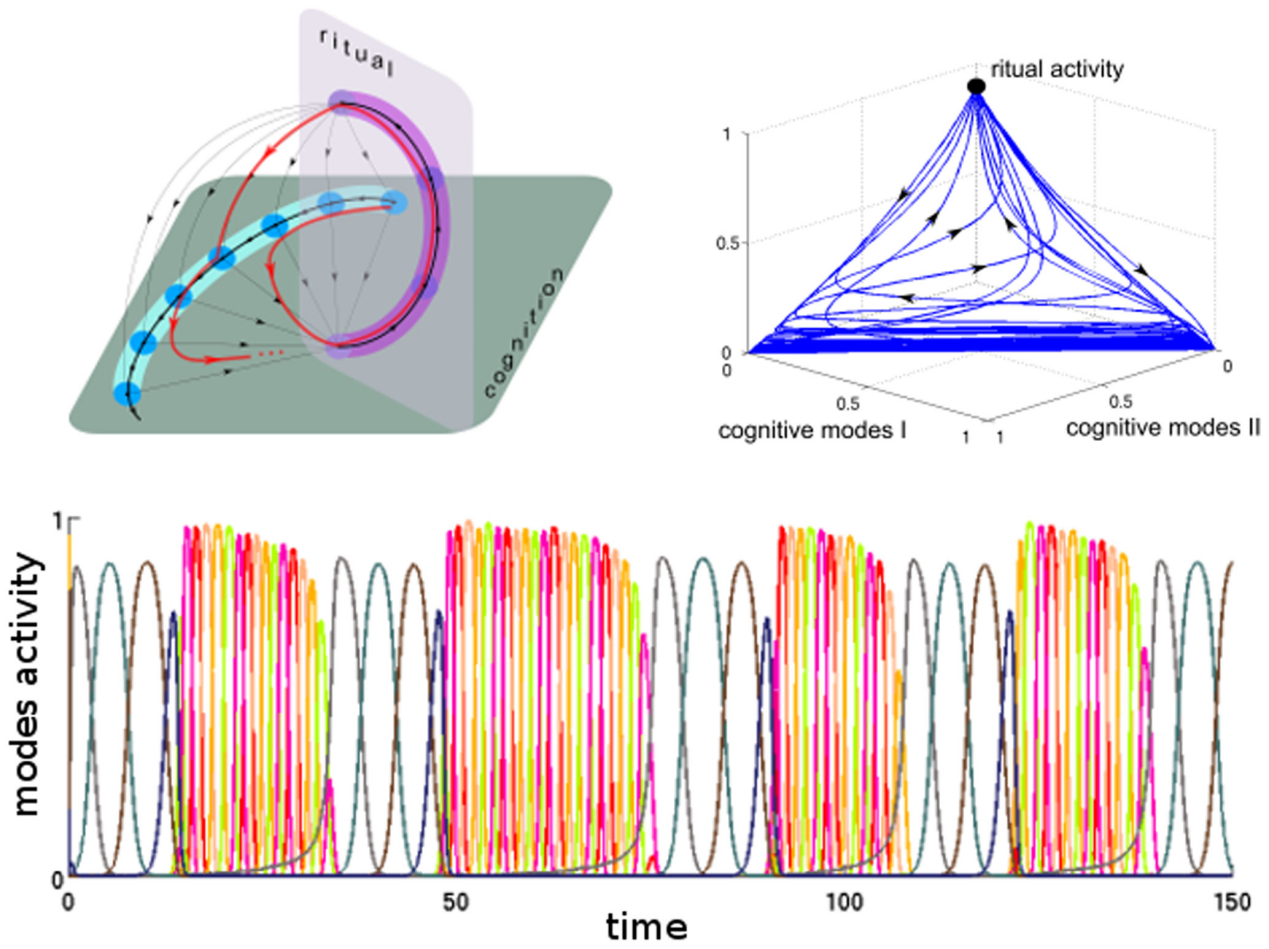
Dynamical representation of Obsessive Compulsive Disorder (OCD). (Top Left) The proposed dynamical image of the OCD. Here, while the cognitive task evolves on stable transients, the dynamics shift towards a dominant intermittent transient sequence (i.e., the ritual) whose initial mode lies on the unstable manifolds of the cognitive saddles. During this ritual, the cognition halts and upon its completion the individual returns to the cognitive process, not necessarily through the last cognitive mode visited. (Bottom) A simulation of OCD by the proposed model. Here the individual performs a “normal” cognitive task represented by the five modes colored yellow-to-red. At certain periods, the individual performs a ritual as illustrated by four dark-colored modes in a prescribed ordered. The system can enter this ritual sequence from any cognitive mode, and, upon completion of the ritual, returns back to the cognitive process via an arbitrary mode. (Top right) A phase portrait of this dynamical behavior.

To model OCD, we introduce a sequence of saddles – that is, metastable states, in the cognitive subspace. Each saddle in this sequence has a two-dimensional unstable manifold, by construction, as described in the Appendix. One of these dimensions forms the separatrix leading to the next cognitive metastable state along a cognitive sequence, whereas the second unstable separatrix targets the emotional saddle that represents the entry to the ritual, which is modeled as a different stable chain of the “emotional” metastable states. The ritual terminates at a saddle that has many unstable separatrices, each yielding to a cognitive mode (see Fig. 4). As a result, the OCD dynamics is represented by a (*N*+*M*)–dimensional transient, which qualitatively distinguishes itself from the normal behavior and from other disorders characterized by a specific instability that leads to uncertainty. The result of modeling predicts that in OCD the interaction of the sequential cognitive activity (e.g., sequential decision making), with emotion is characterized by intermittent dynamical instability. The corresponding dynamical images and phase portraits are represented in Fig. 4.

The OCD model includes enough parameters to describe the competition between the cognitive process and the ritual in great detail. It is possible to make the model ‘non-circular’ by arranging the unstable separatrices of the terminal ritual mode. This adjustment, however, requires clinical data that reveal the return to the cognitive function from the ritual (see also [76]). As one can observe from Figs. 3 and 4, both a panic attack and the OCD have nondeterministic components. However, the mechanisms of the chaotic behavior in these cases are different: the dynamical object that represents the emotion-cognition interaction during a panic attack is transient chaos, whereas a very specific object, which we name “intermittent transient”, describes the emotion-cognition interaction in OCD. Along such an intermittent transient, a chain of metastable cognitive modes is interrupted by the ritualistic behavior characteristic of OCD with an unpredictable returning.

### “Cognitive performance – arousal” interaction. Hysteresis

It is well known that emotions can suddenly switch cognition from one regime to another. Let us use the model (3)–(6) for the analysis of such a phenomenon, i.e., the dependence of cognitive performance on the level of arousal. The popular point of view is that Yerkes-Dodson law dictates an individual's reaction to a stressor: the performance improves with physiological or mental arousal, but only up to a point; when levels of arousal become too high, performance decreases. The process is often illustrated graphically as an inverted U-shaped curve. Despite its plausibility, the Yerkes-Dodson law is difficult to test empirically (see, for example, [77]). Usually the “inverted-U” behavior is realistic enough when the cognitive anxiety is low, i.e., when the human is not worried. Since we are interested in the emotion-cognition interaction, we focus on comparing the model's prediction with previously reported experiments in the case when physiological arousal interacts with cognitive anxiety, i.e. the human is worried to influence his/her performance.

**Figure 5:**
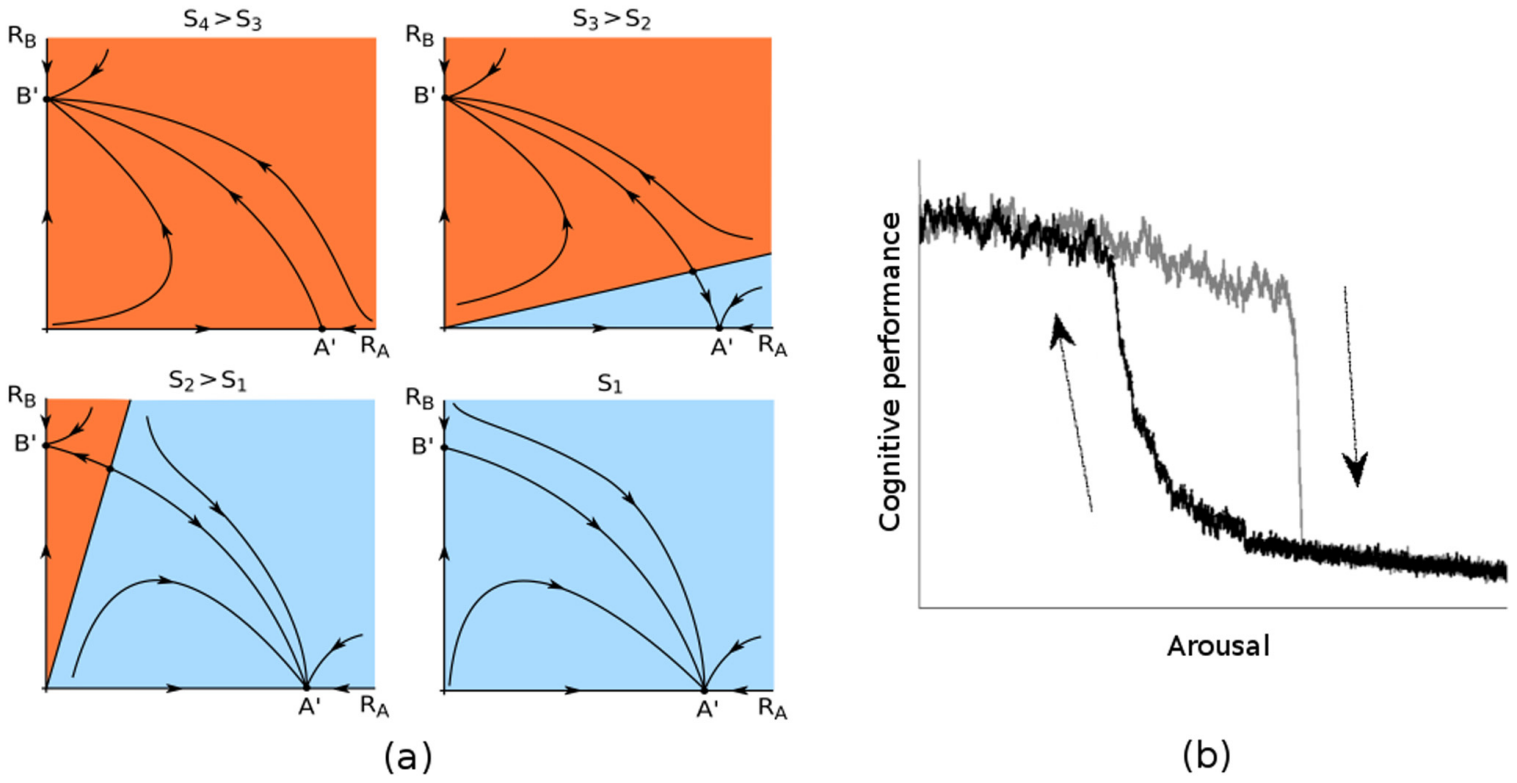
Hysteresis relation between cognitive performance and arousal. (a) The cognitive performance and arousal can have a hysteresis relation. The mechanism responsible from this relation is the indirect (resources) competition among the *R_A_* and *R_B_* variables as introduced in the model. A sequence of phase portraits that corresponds to decreasing stressor (arousal) intensity is shown on the left panel. The attractors are indicated by *A′* and *B′*. Panel (b) illustrates the hysteresis relation observed in the proposed model. The model can successfully reproduce this disorder when (σ,ρ) and (ζ,ξ) pairs satisfy SHC conditions; σ(B) and ζ (*A*,*S*) are non-smooth and large for some *A*, *B*; ζ(*A*,*S*) increases with *S*; and the resource competition is strong with *ϕ_A_*≠*ϕ_B_*.

To the best of our knowledge, currently there is no dynamical model capable of describing an emotion-cognitive hysteresis (see Fig. 5b). Hysteresis phenomenon is well known among sociologists and sport psychologists [78]. To explain it we need to estimate regions or attraction of two equilibria *A′* and B′. Two levels of arousal, where one region (domain) of attraction becomes much larger than the other one and vice versa, determine the hysteresis loop. Our analysis is based on the fact that dynamics of the average levels of emotional and cognitive activities largely coincides with the dynamics of available mental resources (represented by variables *R_A_* and *R_B_* in the model, respectively). The phase portraits of the system in the phase plane (*R_A_*, *R_B_*) are shown in Fig. 5a. One can observe that the area of attraction of state A′ dominates under low arousal levels. As arousal increases, the areas of attraction *A′* and *B′* become comparable, thus manifesting the bistability - further increase in arousal level results in a “monostable” condition *A′* = 0. Since the system “remembers” the initial conditions, the decreasing level of arousal will lead to equilibrium (calm state of mind) only when the area of attraction *A′* greatly exceeds area of attraction *B′*.

## Discussion

In conclusion, we have seen that the suggested coarse-grain model based on the formulated principles is potentially very powerful. This model operates on the activities of correlated neuronal groups distributed in the brain, abstractly termed mental modes. In principle, it is possible to describe the anatomical structure of many cognitive and emotion modes, based on imaging data that are available today. However, from the practical point of view, at neuronal level it still may not be helpful to make a direct connection of our phenomenological model with neurobiology. This is because it is currently impossible to determine the functional connections between different modes. Thus, at just glance, our approach (based on the observation of behavioral transformations) seems like the only reasonable one presently. A key object in the model dynamics is a stable transient realized through a reproducible serial order of metastable states. As a generic dynamical phenomenon, which is rare in simple systems yet common in complex ones, the sequential switching among metasable states can provide concise and constructive formulations in a variety of mental problems [79]. The prototype dynamical models are widely accepted in neuroscience [80]. The key physiological mechanism underlying the winnerless competition (WLC) and sequential switching in the brain is nonsymmetric inhibition, which is known to exist in neural systems from micro- to macroscopic levels [81]–[85]. WLC is observed in biological systems, in particular, in two *in vivo* experiments, e.g., gustatory and olfactory sensory systems [23], [86], [87].

According to the standard definition, a dynamical system is a model of a real system that undergoes a time evolution from the initial moment to infinity. The cortex, strictly speaking, is not a dynamical system under this definition. However, many mental functions demonstrate dynamical features and can be described by appropriate dynamical models over finite intervals of time. Furthermore, the brain is somehow capable of coordinating the results of interrupted dynamical activities as a “sequence of sequences” (see also [88]). One can hypothesize that, in such cases, mental modes coordinate their activities in accordance with the universal principles that we have already discussed in this paper. To handle the uncertainty that is typical for the interrupted sequences one needs additional principles - presumably the minimum predictive error, the minimum information principle [89], or the free energy principle [90].

## Supporting Information

File S1 (0.06 MB DOC)

## References

[1] James W (1890). The principles of psychology.

[2] Poincaré H (1905). The Value of Science..

[3] Yuste R (2005). The cortex as a central pattern generator.. Nat Rev Neurosci.

[4] Reichle ME (2010). Two views of brain function.. Trends in Cognitive Sciences.

[5] Lashley KS, Jeffress LA (1951). The problem of serial order in behavior.. Cerebral mechanisms in behavior (pp 112–131).

[6] Port RF, van Gelder T (1995). Mind as motion: explorations in the dynamics of cognition description.

[7] Schurger A, Pereira F, Treisman A, Cohen JD (2010). Reproducibility distinguishes conscious from nonconscious neural representations.. Science.

[8] Temam R (1988). Infinite dimensional dynamical systems in mechanics and physics.

[9] Hopfield JJ (1982). Neural networks and physical systems with emergent collective computational abilities.. Proc Natl Acad Sci U S A.

[10] Cohen MA, Grossberg S (1983). Absolute stability and global pattern formation and parallel memory storage by competitive neural networks.. IEEE Trans Syst Man Cybern.

[11] Wills TJ, Lever C, Cacucci F, Burgess N, O'Keefe J (2005). Attractor dynamics in the hippocampal representation of the local environment.. Science.

[12] Afraimovich V, Zhigulin V, Rabinovich M (2004). On the origin of reproducible sequential activity in neural circuits.. Chaos.

[13] Abeles M, Bergman H, Gat I, Meilijson I, Seidemann (1995). Cortical activity flips among quasi-stationary states.. Proc Nat Acad Sci U S A.

[14] Bressler SL, Kelso JAS (2001). Cortical coordination of dynamics and cognition.. Trends in Cognitive Sciences.

[15] Oullier O, Kelso JAS (2006). Neuroeconomics and the metastable brain.. Trends Cogn.

[16] Werner G (2007). Metastability, criticality and phase transitions in brain and its models.. Biosystems.

[17] Friston KJ (1997). Transients, metastability, and neuronal dynamics.. NeuroImage.

[18] Friston KJ (2000). The labile brain. I. neuronal transients and nonlinear coupling.. Philos Trans R Soc Lond B Biol Sci.

[19] Ito J, Nikolaev AR, van Leeuwen C (2007). Dynamics of spontaneous transitions between global brain states.. Hum Brain Mapp.

[20] Gros C (2007). Neural networks with transient state dynamics.. New Journal of Physics.

[21] Fingelkurts AA, Fingelkurts AA (2006). Timing in cognition and EEG brain dynamics: discreteness versus continuity.. Cogn Process.

[22] Chen Y, Bressler SL, Ding M (2009). Dynamics on networks: assessing functional connectivity with Granger causality.. Comput Math Organ Theo.

[23] Jones LM, Fonranini A, Sadacca BF, Miller P, Katz DB (2007). Natural stimuli evoke dynamic sequences of states in sensory cortical ensembles.. Proc Nat Acad Sci U S A.

[24] Rabinovich MI, Huerta R, Laurent G (2008). Transient dynamics for neural processing.. Science.

[25] Kubota S, Sakai K (2002). Relationship between obsessive-compulsive disorder and chaos.. Medical Hypotheses.

[26] Zor R, Hermesh H, Szechtman H, Eilam D (2007). Turning order into chaos through repetition and addition of elementary acts in obsessive-compulsive disorder (OCD).. World J Biol Psychiatry, Jul.

[27] Schraagen JM, Chipman SF (2000). Cognitive task analysis.

[28] Hollnagel E (2003). Handbook of cognitive task design.

[29] Price DD, Barreil JE, Barrell JJ (1985). A quantitative-experimental analysis of human emotions.. Motivation and Emotion.

[30] Scherer KR (1993). Neuroscience projections to current debates in emotion psychology.. Cognition and Emotion.

[31] Thagard P, Aubie B (2008). Emotional consciousness: a neural model of how cognitive appraisal and somatic perception interact to produce qualitative experience.. Consciousness and Cognition.

[32] Pessoa L, Padmala S (2005). Quantitative prediction of perceptual decisions during near-threshold fear detection.. Proc Natl Acad Sci U S A.

[33] Lee JH (1999). Test anxiety and working memory.. Journal of Experimental Education.

[34] Fales CL, Barch D, Burgess GC, Schaefer A, Mennin DS (2008). Anxiety and cognitive efficiency: Differential modulation of transient and sustained neural activity during a working memory task.. Cognitive, Affective, & Behavioral Neuroscience.

[35] Landau LD, Lifschitz EM (1959). Fluid Mechanics, Course of Theoretical Physics Vol. 6 (second edition).

[36] Moran RJ, Kiebel SJ, Stephan KE, Reilly RB, Daunizeau J (2007). A neural mass model of spectral responses in electrophysiology.. NeuroImage.

[37] Zavaglia M, Astolfi L, Babiloni F, Ursino M (2006). A neural mass model for the simulation of cortical activity estimated from high resolution EEG during cognitive or motor tasks.. Journal of Neuroscience Methods.

[38] Nunez PL, Srinivasan B (2005). Electric fields of the brain: The neurophysics of EEG, 2^nd^ ed.

[39] Friston K, Phillips J, Chawla D, Buechel C (2000). PCA: characterizing interactions between modes of brain activity.. Philos Trans R Soc Lond B Biol Sci.

[40] Koenig T, Marti-Lopez F, Valdes-Sosa O (2001). Topographic time-frequency decomposition of the EEG.. NeuroImage.

[41] McKeown MJ, Makeig S, Brown GG, Jung TP, Kindermann (1998). Analysis of fMRI Data by Blind Separation Into Independent Spatial Components.. Human Brain Mapping.

[42] Lane RD, Pollermann BZ, Barrett LF, Salovey P (2002). Complexity of emotion representations.. The wisdom in feeling: psychological processes in emotional intelligence.

[43] Rabinovich MI, Varona P, Selverston AI, Abarbanel HDI (2006). Dynamical Principles in Neuroscience.. Reviews of Modern Physics.

[44] Huerta R, Rabinovich MI (2004). Reproducible sequence generation in random neural ensembles.. Phys Rev Lett.

[45] Lotka AJ (1956). Elements of mathematical biology.

[46] Muezzinoglu MK, Tristan I, Huerta R, Afraimovich V, Rabinovich MI (2009). Transients versus attractors in complex systems.. Int J Bifurcation Chaos.

[47] Varona P, Levi R, Arshavsky YI, Rabinovich MI, Selverston AI (2004). Competing sensory neurons and motor rhythm coordination.. Neurocomputing.

[48] Huber MT, Braun HA, Krieg JC (1999). Consequences of deterministic and random dynamics for the course of affective disorders.. Biological Psychiatry.

[49] Huber MT, Braun HA, Krieg JC (2000). Effects of noise on different disease states of recurrent affective disorders.. Biological Psychiatry.

[50] Krumhansl CL (2000). Rhythm and pitch in music cognition.. Psychological Bulletin.

[51] Cureton RD (1994). Rhythmic cognition and linguistic rhythm.. Journal of Literary Semantics.

[52] Buckner RL, Andrews-Hanna JR, Schacter (2008). The brain's default network: anatomy, function, and relevance to disease.. Annals of the New York Academy of Sciences.

[53] Fox MD, Snyder AZ, Vincent JL, Corbetta M, Van Essen DC (2005). The human brain is intrinsically organized intodynamic, anticorrelated functional networks.. Proc Nat Acad Sci U S A.

[54] Tomasi D, Volkow ND (2010). Functional connectivity density mapping.. Proc Natl Acad Sci USA.

[55] Magor L, Lorincz F, Geall Y, Bao V, Crunelli SW (2009). ATP-Dependent Infra-Slow (,0.1 Hz) Oscillations in Thalamic Networks.. PLoS ONE.

[56] Taylor KS, Seminowicz DA, Davis KD (2009). Two systems of resting state connectivity between the insula and cingulate cortex.. Hum Brain Mapp.

[57] Buckner RL (2010). Human functional connectivity: New tools, unresolved questions.. PNAS.

[58] Salvador R, Suckling J, Schwarzbauer C, Bullmore E (2005). Undirected graphs of frequency-dependent functional connectivity in whole brain networks.. Philosophical Transaction of the Royal Society B.

[59] Broyd SJ, Demanuele C, Debener S, Helps SK, James CJ (2009). Default-mode brain dysfunction in mental disorders: A systematic review.. Neurosci Biobehav Rev.

[60] Mannell MV, Franco AR, Calhoun VD, Cañive JM, Thoma RJ (2010). Resting state and task-induced deactivation: a methodological comparison in patients with schizophrenia and health controls.. Human Brain Mapping.

[61] Fornito A, Bullmore ET (2010). What can spontaneous fluctuations of the blood oxygenation-level-dependent signal tell us about psychiatric disorders?. Current Opinion in Psychiatry.

[62] Baliki MN, Geha PY, Apkarian AV, Chialvo DR (2008). Beyond feeling: chronic pain hurts the brain, disrupting the default-mode network dynamics.. Journal of Neuroscience.

[63] Ploghaus A, Tracey I, Gati JS (1999). Dissociating pain from its anticipation in the human brain.. Science.

[64] Nutt DJ (2001). Neurobiological mechanisms in generalized anxiety disorders.. Journal of Clinical Psychiatry.

[65] Masterpasqua F, Perna PA (1997). The psychological meaning of chaos: Translating theory into practice.

[66] Katerndahl D, Ferrer R, Best R, Wang C-P (2007). Dynamic patterns in mood among newly diagnosed patients with major depressive episode or panic disorder and normal controls.. Prim Care Companion J Clin Psychiatry.

[67] Kubota S, Sakai K (2002). Relationship between obsessive-compulsive disorder and chaos.. Medical Hypotheses.

[68] Zor R, Hermesh H, Szechtman H, Eilam D (2007). Turning order into chaos through repetition and addition of elementary acts in obsessive-compulsive disorder (OCD).. World J Biol Psychiatry, Jul.

[69] Ott E (1993). Chaos in dynamical systems.

[70] Sabelli HC, Carlson-Sabelli L, Patel M, Levy A, Diez-Martin J, Robertson R, Combs A (1995). Anger, fear, depression, and crime: Physiological and psychological studies using the process method.. Chaos Theory in Psychology and the life sciences.

[71] Callahan J, Sashin JI (1990). Predictive models in psychoanalysis.. Behavioral Science.

[72] Gustafson JP, Meyer M (2004). A non-linear model of dynamics: a case of panic.. Psychodynamic Practice.

[73] Huppert JD, Franklin ME (2005). Cognitive behavioral therapy for obsessive-compulsive disorder: an update.. Curr Psychiatry Rep.

[74] Hollander E, Kim S, Khanna S, Pallanti S (2007). Obsessive-compulsive disorder and obsessive-compulsive spectrum disorders: Diagnostic and dimensional issues.. CNS Spectr.

[75] Rolls ET, Loh M, Deco G (2008). An attractor hypothesis of obsessive-compulsive disorder.. Eur J Neursci.

[76] McClure SM, Botvinick MM, Yeung N, Greene JD, Cohen JD, Gross JJ (2007). Conflict monitoring in cognition–emotion competition.. Handbook of emotion regulation (pp 204–226).

[77] Moran AP (2004). Sport and exercise psychology: a critical introduction, Routledge, Canada.

[78] Jones JG, Hardy L (1990). Stress and performance in sport.

[79] Afraimovich V, Tristan I, Huerta R, Rabinovich MI (2008). Winnerless competition principle and prediction of the transient dynamics in a Lotka-Volterra model.. Chaos.

[80] Wilson HR, Cowan JD (1973). A mathematical theory of the functional dynamics of cortical and thalamic nervous tissue.. Kybernetik.

[81] Aron AR (2007). The neural basis of inhibition in cognitive control.. The Neuroscientist.

[82] Kelly AMC, Uddin LQ, Biswal BB, Castellanos FX, Milhan MP (2008). Competition between functional brain networks mediates behavioral variability.. NeuroImage.

[83] Jaffard M, Longcamp M, Velay JL, Anton JL, Roth M (2008). Proactive inhibitory control of movement assessed by event-related fMRI.. NeuroImage.

[84] Buzsaki G, Kaila K, Raichle M (2007). Inhibition and brain work.. Neuron.

[85] Ponzi A, Wickens J (2010). Sequentially switching cell assemblies in random inhibitory networks of spiking neurons in the striatum.. Journal of Neuroscience.

[86] Mazor O, Laurent G (2005). Transient dynamics versus fixed points in odor representations by locust antennal lobe projection neurons.. Neuron.

[87] Stopfer M, Jayaraman V, Laurent G (2003). Intensity versus identity coding in an olfactory system.. Neuron.

[88] Kiebel SJ, von Kriegstein K, Daunizeau J, Friston KJ (2009). Recognizing sequences of sequences.. PLoS Computational Biology.

[89] Globerson A, Stark E, Vaadia E, Tishby N (2009). The minimum information principle and its application to neural code analysis.. Proc Natl Acad Sci U S A.

[90] Friston KJ, Daunizeau J, Kilner J, Kiebel SJ (2010). Action and behavior: a free-energy formulation.. Biol Cybern.

