# Supplementary material for: Dynamical Principles of Emotion-Cognition Interaction: Mathematical Images of Mental Disorders
Source: PLoS One. 2010 Sep 21;5(9):e12547. doi: 10.1371/journal.pone.0012547 (PMC2943469; doi:10.1371/journal.pone.0012547)
Supplement: File S1 — (0.06 MB DOC) [file pone.0012547.s001.doc]

**Supporting Information**

**Structural Stability of Transients and Generalized Lotka-Volterra Model**

We consider *n* saddles as the basis of our construction of a reproducible transient behavior. A saddle is a stationary point that possess both stable (attractive) and unstable (repellant) manifolds. This distinction is made based on the sign of Lyapunov exponents (eigenvalues) corresponding to these manifolds. The transient nature of the system is due to the organization of the saddles in such a way that each saddle’s unstable (i.e., escape) manifold corresponds to a stable manifold of the following saddle. To enlighten the conditions on the stability of this sequence of metastable states, we consider an elementary phase volume in the neighborhood of each saddle that is compressed along the stable separatrices and stretched along the unstable separatrix. Let us order the eigenvalues of saddle *i* as

,

which imposes only one unstable manifold to each saddle. The number

$$\nu_{i}=\frac{-\mathrm{Re}\left\{\lambda_{2}^{i}\right\}}{\lambda_{1}^{i}}$$

is called the saddle value. If ν*_i_*> 1, then the compression along the stable manifolds dominates the stretching along the unstable manifold, and the saddle is called as a dissipative saddle. If all saddles in the sequence are dissipative, then the trajectories in their vicinity cannot escape from the vicinity of the chain, providing stability. It possible estimate the time spent around each saddle by

$$T_{i}=\frac{1}{\lambda_{1}^{i}}\ln\frac{1}{\left|\eta\right|}$$

|η| denoting the magnitude of the noise level on the vector field.

A normal form of competition among *n* agents is the Generalized Lotka-Volterra (GLV) equations given by

$$\tau\frac{d}{dt}x_{i}\left(t\right)=x_{i}\left(t\right)\left[\mu_{i}\left(E\right)-\sum_{j=1}^{n} \phi_{ij}\left(E\right)x_{i}\left(t\right)\right]+x_{i}\left(t\right)\eta\left(t\right),$$

Here *t* denotes the time, *x_i_* ≥ 0 is the *i*-th competing agent, *E* is the input that captures all (known) external effects on the competition, τ is the time-constant, µ*_i_*’s are the increments (or, stimulations) that represent the resources available to the competitor *i* to prosper, ϕ*_ij_* is the competition matrix, and η(*t*) is a multiplicative noise perturbing the system.

Depending on configuration of parameters, this model exhibits a vast variety of behaviors. When connections are nearly symmetric, i.e., ϕ*_ij_* ≈ ϕ*_ji_*, two or more stable states can co-exist, yielding a multi-stable behavior The initial conditions determine which of the stable states sets in. With strongly non-symmetric connections there appear heteroclinic or limit cycles, stable sequence of metastable states and dynamical chaos. We also investigated a specific kind of dynamical chaos, where the order of the switching is deterministic and stable, but the life-time of the metastable states is random [30]. Reproducibility of transient dynamics, that is the preservation of the order of metastable states despite the irregularity in timing, can be quite interesting for understanding of cognitive processes and analysis of the observed data.

In the GLV model, the saddle value condition for stability can be written by the following inequalities on system parameters [12]:

$\frac{\mu_{i_{k-1}}}{\mu_{i_{k}}}<\phi_{i_{k-1}i_{k}}<\frac{\mu_{i_{k-1}}}{\mu_{i_{k}}}+1$ , *k* = 2,…,*n*  (A1)

$\frac{\mu_{i_{k+1}}}{\mu_{i_{k}}}-1<\phi_{i_{k+1}i_{k}}<\frac{\mu_{i_{k+1}}}{\mu_{i_{k}}}$ , *k* = 1,…,*n*–1  (A2)

$\phi_{ii_{k}}>\phi_{i_{k-1}i_{k}}+\frac{\mu_{i}-\mu_{i_{k-1}}}{\mu_{i_{k}}}+1$ , *k* = 2,…,*n*  (A3)

where {*i*_1_,…, *i_k_*,… *i_n_*} is the sequence of saddles and the third inequality (A3) applies for all *i* other than *i_k-1_*, *i_k_*, *i_k_*_+1_.

The inequalities (A1)-(A3) introduce a single unstable manifold to each saddle in the chain; all other *n*-1 manifolds are stabilized due to the coverage of (A3). Additional unstable manifolds can be introduced to any saddle by imposing new inequalities in the form of (A1) and (A2) and by relaxing (i.e., removing) some inequalities of the form (A3). To illustrate this case, without loss of generality, we consider the chain {*i*_1_, *i_n+1_*, …, *i_n+l_*} to co-exist with the previous chain now in the augmented *n* + *l* dimensional state-space. The new situation requires that the saddle labeled by *i*_1_now has a two-dimensional unstable manifold spanned by the separatrices from saddle *i*_1_ toward the saddles *i*_2_ and *i_n_*_+1_.The realize the two chains, the inequalities (A1) and (A2) are imposed for each transition (or, separatrix) observed in the two sequences; note that the two unstable separatrices of the joint saddle do not cause any contradiction by (A1) and (A2). However, the stability condition (A3) should now run for a reduced number of indices, namely for all *i* other than *i*_1_, *i*_2_, *i_n_*_+1_, *i_n_*_+2_.
